# The quest for a better insight into physiology of fluids and barriers of the brain: the exemplary career of Joseph D. Fenstermacher

**DOI:** 10.1186/2045-8118-12-1

**Published:** 2015-01-12

**Authors:** Adam Chodobski, Jean-François Ghersi-Egea, Charles Nicholson, Tavarekere N Nagaraja, Joanna Szmydynger-Chodobska

**Affiliations:** Department of Emergency Medicine, Neurotrauma and Brain Barriers Research Laboratory, The Warren Alpert Medical School of Brown University, Coro Center West, Room 112, 1 Hoppin Street, Providence, RI 02903 USA; Blood-Brain Interface Group, Oncoflam Team and BIP Platform INSERM U 1028, CNRS UMR5292 Lyon Neuroscience Research Center, Faculté de Médecine RTH Laennec, Rue Guillaume Paradin, Cedex 08, 69372 Lyon, France; Department of Neuroscience and Physiology, NYU School of Medicine, MSB 460, 550 First Avenue, New York, NY 10016 USA; Department of Anesthesiology, Henry Ford Hospital, 2799 West Grand Blvd., Detroit, MI 48202-2689 USA

**Keywords:** Cerebrospinal fluid, Brain extracellular space, Ventriculocisternal perfusion, Diffusion, Radiolabelled tracers, Cerebral blood flow, MRI contrast agent, Tumour therapy

## Abstract

In June 2014 Dr. Joseph D. Fenstermacher celebrated his 80th birthday, which was honored by the symposium held in New London, NH, USA. This review discusses Fenstermacher’s contribution to the field of fluids and barriers of the CNS. Specifically, his fundamental work on diffusion of molecules within the brain extracellular space and the research on properties of the blood–brain barrier in health and disease are described. Fenstermacher’s early research on cerebrospinal fluid dynamics and the regulation of cerebral blood flow is also reviewed, followed by the discussion of his more recent work involving the use of magnetic resonance imaging.

## Introduction

In June of 2014 a symposium was held in New London, NH, USA to celebrate the 80th birthday of Dr. Joseph D. Fenstermacher and to highlight his exceptional contribution to the field of fluids and barriers of the CNS. Over the past 50 years remarkable progress has been made in our knowledge of the function of the blood–brain barrier (BBB) and blood-cerebrospinal fluid barrier (BCSFB), and physiology of extracellular fluids in the brain. The work of Joseph Fenstermacher, his students, and his colleagues was instrumental in providing the basis for our current understanding of these aspects of brain function.

In this article, we will discuss Fenstermacher’s contribution to the field, and also reflect on how his early work and the experimental tools at his disposal allowed him to describe the physiological phenomena. We also show that with the help of modern research technologies, these can now be confirmed with an improved understanding. This review will start with a section written by Charles Nicholson introducing the reader to Fenstermacher’s early work on diffusion of radiotracers within the brain extracellular space (ECS) and the assessment of its volume fraction. The next section, written by Adam Chodobski and Jean-François Ghersi-Egea, will focus on early investigations of cerebrospinal fluid (CSF) formation and space, followed by early studies of the regulation of cerebral blood flow (CBF). The final part, written by Tavarekere Nagaraja, will be devoted to the last seventeen years of Fenstermacher’s work during which he and his colleagues validated modern technologies such as magnetic resonance imaging (MRI) by concurrently employing previously established radioisotopic techniques.

## Review

### Ventriculocisternal perfusion applied to the study of diffusion in ECS

The ECS is the totality of the narrow gaps that separate adjacent cells of the brain. In the 1950’s and 1960’s there was much controversy about this domain. The width of the ECS was thought to be much less than a micrometer; nonetheless, because a tiny atmosphere of ECS surrounds each cell membrane and there are a huge number of cells, the fraction of brain tissue occupied by ECS (volume fraction) could be appreciable. The problem in the early days was to assign a numerical value to the volume fraction and the numbers obtained ranged widely. On one side were the electron microscopists who obtained different, and usually small, values depending on how they euthanized the animals and fixed the tissue, because the width of the ECS is exceedingly sensitive to ischemia and the water content of the cells [1]. On the other side were the physiologists who bathed a volume of tissue in a small radiotracer that was supposed to permeate only the ECS, and then measured how much had equilibrated with the total tissue volume [2]; unfortunately, many tracers used are far from impermeable.

**Figure 1:**
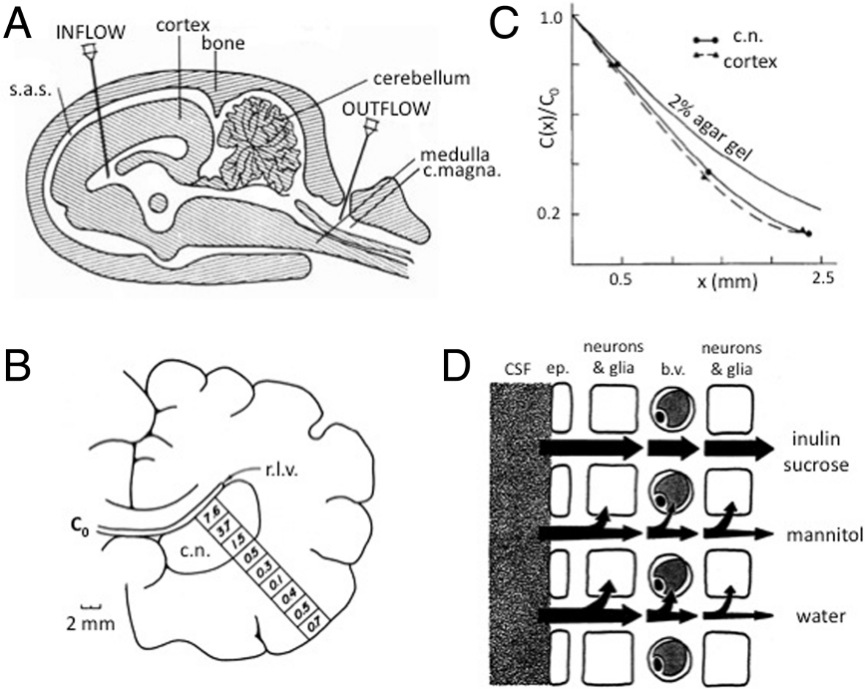
**Diffusion measurements using ventriculocisternal perfusion. A**. Diagram of ventriculocisternal perfusion system in sagittal view. Physiological saline containing radiotracer enters via the INFLOW cannula, flows predominantly through the lateral, III, and IV ventricles and leaves via the OUTFLOW cannula (Modified from [8]). **B**. Typical locations of samples of radiotracer recovered from fixed dog brain in coronal section. Right lateral ventricle (r.l.v.) is flushed with radiolabelled inulin (at concentration *C*_0_, see Eq. (1)). Numbers in each square represent percentage of *C*_0_ recovered after 3–5 hours through caudate nucleus (c.n.) and cortex. Note that tracer count increases towards cortical surface because some tracer perfuses the subarachnoid space, (modified from [3]). **C**. Relative concentration profiles in cat brain after 132 minutes of ventriculocisternal perfusion with radiolabelled inulin. Profiles shown in caudate nucleus and cortex together with profiles in 2% agar gel at the same time for comparison. Note that diffusion in the brain is more hindered than in agar. From curves like these the effective diffusion coefficients may be calculated using Eq. (1), (modified from [7]). **D**. Diagram of pathways that a molecule may follow after entering the brain from the CSF at a ventricular surface. Inulin and sucrose remain in the ECS but mannitol enters neurons and glia to some extent and also crosses the blood–brain barrier into blood vessels (b.v.). Water is not confined to the ECS but exchanges with other compartments relatively freely, (modified from [6]. Abbreviations: *b.v.*: blood vessel; *c. magna*: cisterna magna; *c.n.*:caudate nucleus; *ep*: ependymal cell; *r.l.v.*: right lateral ventricle; *s.a.s.*: subarachnoid space.

As is often the case when the topic engenders heated discussion, the resolution required a better technique and this duly appeared in the form of ventriculocisternal perfusion with radiolabeled inulin (Figure 1A). Rall, Oppelt and Patlak [3] used this method to establish the volume fraction of the ECS in the caudate nucleus of mongrel dogs. They based their results on tissue samples taken at different time points from the first few millimeters from the ventricle. Because they took samples all the way through the thickness of the caudate and cortex, the figure in their paper clearly shows that they had established a gradient of concentration (Figure 1B). Such a gradient is a characteristic of diffusion and a measurement of the gradient has the potential to yield the effective diffusion coefficient for the substance of choice in the tissue. A subsequent paper by Oppelt and Rall [4] exploited this concept to make some of the first estimates of volume fraction and effective diffusion coefficients, primarily using inulin and sucrose in several brain regions and animal species, including: “live dog, dead dog, live cat, dead cat, live green monkey, dead green monkey, live rhesus monkey, dead rhesus monkey”.

After the ventriculocisternal perfusion method became established there was a need to refine it and expand the work. This is where Fenstermacher appears in the publications with this method. In fact, he had made an earlier attempt to measure ECS volume fraction using subarachnoid perfusion but the results gave a low value, which even the authors themselves questioned [5]. The paper by Fenstermacher, Rall, Patlak and Levin [6] is primarily about the application of the ventriculocisternal perfusion method and it further established that sucrose and inulin were good ECS molecules for studying diffusion in the ECS (Figure 1D). Levin, Fenstermacher and Patlak [7] used the technique to make extensive measurements of ECS volume fraction in rabbits, cats, dogs and monkeys. They concluded that the volume fraction (*α*) in the cerebral cortex, averaged across all four species, was 19.7% for sucrose and 18.3% for inulin. It may be noted that the volume fraction values are close to those reported with more recent ion-selective microelectrode measurements that will be described below. These investigators were also able to make a rough estimate of the effective diffusion coefficients for these two substances based on the depth profile of concentration (Figure 1C).

The parameters of the ventriculocisternal method had become established (Figure 1A; [8]); however it took a few years to realize fully the potential of the approach. This became evident in two papers, one published in 1974 and the other in 1975; indeed, in these two papers, we are no longer talking about just diffusion but the method has been broadened to include molecular transport at the ECS interfaces. In Fenstermacher, Patlak and Blasberg [9] a new level of sophistication was reached: working on the dogfish at the Mount Desert Island Biological Laboratory, the ventriculocisternal perfusion method was extended to determine the transfer constants at the ependymal layer of the ventricles and at the capillary level of the BBB while taking into account possible equilibration between extra- and intracellular spaces. This short paper did not fully describe the equations involved but these appear in detail in Patlak and Fenstermacher [10], which may be one of the crowning achievements of this era of diffusion studies.

The 1975 study returned to the dog and focused on caudate nucleus and cortex, measuring all the parameters outlined in the 1974 paper. In addition, careful measurements confirmed just how much the ECS hindered the movement of a small extracellular molecule, like sucrose, showing that its effective diffusion coefficient (*D*_effective_) was about 45% of the value in water. The hindrance to diffusion was measured by calculating the tortuosity value, *λ*, defined as *λ* = (*D*/*D*_effective_)^1/2^ where *D* was the free diffusion coefficient. The value of *λ* was calculated from the one-dimensional solution to the diffusion equation, which in its simplest form (see Eq. 19 in [10]) is:
1

where *C*(*x,t*) is the concentration at depth *x* and time *t* and *C*_0_ is concentration in the perfusate. The function ‘erfc’ is the complementary error function.

This approach gave a *λ*-value for sucrose of about 1.5 [10]. Effective diffusion coefficients for inulin, sucrose and sodium are summarized in Fenstermacher and Patlak [11] confirming that the tortuosity established with the first two molecules was 1.5. Sodium yielded a tortuosity of 1.8, doubtless reflecting the fact that this cation is not wholly confined to the ECS. Again, the tortuosity values established with the two neutral molecules have been confirmed by more recent measurements with ion-selective microelectrodes. Fenstermacher and Patlak [11] also provided an interesting discussion of bulk flow in brain tissue – a topic that has been under discussion for many years [12], and which has become prominent again lately [13, 14].

By 1975, it was also becoming clear that radiolabeled sucrose was the probe of choice for a molecule that remains predominantly in the ECS (inulin is less reliable). In many practical situations, however, one wants to study molecules that penetrate cells or move across the BBB (Figure 1D). One example is molecules that are used for chemotherapy in brain tissue. In one of several studies, Blasberg, Patlak and Fenstermacher [15] applied ventriculocisternal perfusion to the study of the diffusion properties of five different chemotherapeutic drugs in monkey brain and concluded that they differ in their distribution space and capillary permeability thus showing that different application regimens will be required for effective drug application. The distribution space is defined as the space that the molecules actually occupy; depending on the technique used to make the measurement, the volume fraction obtained may exceed unity if molecules are actively accumulated in cells. In 1976, a series of studies with John Kessler expanded the ventriculocisternal perfusion methodology to subarachnoid perfusion in monkey spinal cord, e.g. [16], paving the way for better drug application to this important part of the CNS. The studies on the spinal cord lacked some of the precision seen in the cortex, doubtless because the small cross-section of the spinal cord does not lend itself well to perfusion studies.

This brief survey of Fenstermacher’s considerable impact on the quantitative analysis of diffusion has recognized his pioneering contributions but omitted many relevant papers. For a more extensive, though also not complete, summary of the radiotracer work see Fenstermacher and Kaye [17].

### The point-source paradigm for ECS diffusion measurements

One of the advantages of ventriculocisternal perfusion with radiotracers is that it may be used with a wide variety of substances subject only to the condition that they can be formulated as a radiotracer. There are significant disadvantages to the method, however. Only one time-point can be obtained per animal and the blocks of tissue harvested to determine concentration must be sufficiently large to achieve an accurate reading, which limits spatial resolution and promotes the use of large brains and consequently, large animals, such as dogs. Only tissue fairly close to the perfusion surface may be analyzed and must broadly conform to a one-dimensional diffusion problem. This also implies that the tissue under analysis must be homogeneous in structure. Finally, radioactive substances must be used with their attendant practical issues.

**Figure 2:**
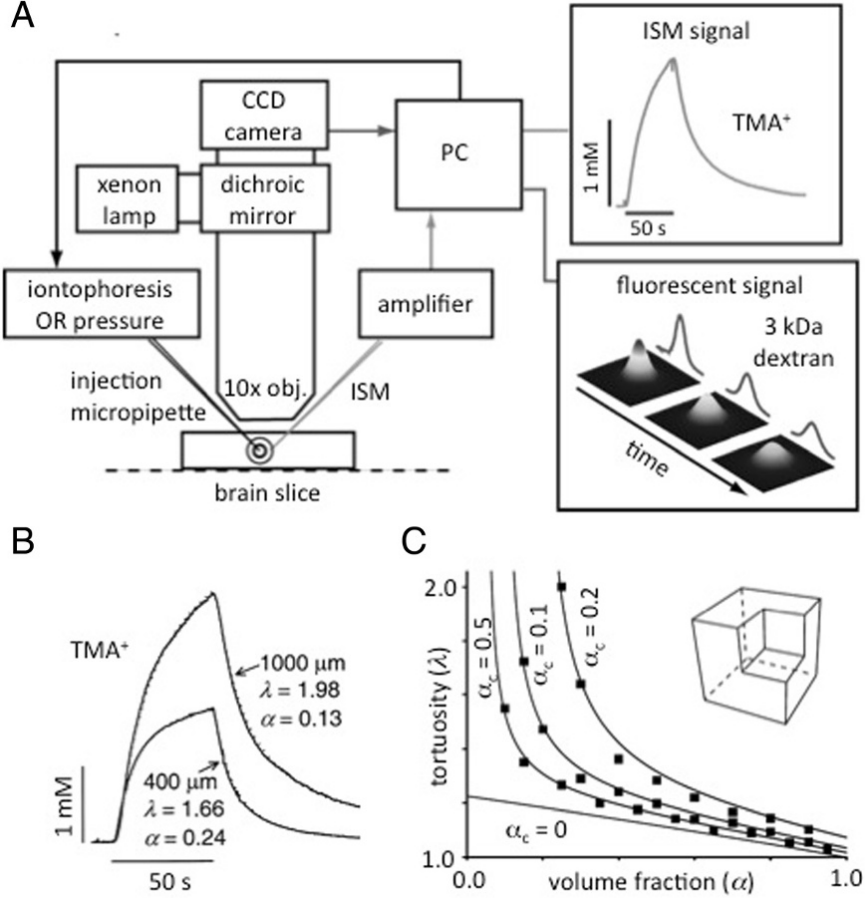
**Diffusion measurements using point-source paradigm and a model of extracellular space. A**. Recording setup for diffusion measurements using RTI and IOI methods. Either a brain slice (shown here) or anesthetized animal rests on the stage of a compound microscope. For RTI, TMA^+^ is iontophoresed from the injection micropipette and detected with an ISM about 100 μm away. The resulting voltage-versus-time curve is digitized and converted to concentration-versus-time using a personal computer (PC). By fitting Eq. (2) or a related equation, *D*_effective_ (and hence *λ*) and *α* are measured. For IOI, a fluorescent molecule, (3 kDa MW dextran here) is pressure injected and a time-series of images captured using a 10× objective (obj.) and a charge-coupled device (CCD) camera. A solution of the diffusion equation (similar to Eq. (3)) is fitted to the intensity profiles and *D*_effective_ extracted. **B**. Diffusion curves with TMA^+^ in normal and thick rat cortical slices. Separation between source and ISM was 100 μm. In normal (400 μm) slices typical values of *λ* and *α* were measured. In thick slices (1000 μm), representing ischemic brain tissue, *λ* increased and *α* decreased, (modified with permission from [24]). **C**. Monte Carlo computer simulation of diffusion in 3D medium containing concave dead spaces. Brain tissue modeled from cubes surrounded by a thin atmosphere of ECS and with a cubic cavity at one corner. This cavity was surrounded by three cubes and associated ECS creating a dead-space. Volume fraction was varied from 0.05 to 0.90 with *α*_C_ equal to 0.05, 0.10, and 0.20. Filled symbols show simulation results and continuous lines came from Eq. (4) with *β* = 3. Modified Maxwell relationship (Eq. (4) with *α*_C_ = 0) derived from simulations using cells without cavities also shown (modified with permission from [34]).

Some of the disadvantages of the radiotracer method were overcome through the introduction of the ‘point-source paradigm’ by Charles Nicholson and co-workers. The idea is simply to release a substance from a micropipette and then measure the concentration as a function of time and distance, fit the appropriate solution of the diffusion equation and extract *α* and *λ* (Figure 2A). The first detailed implementation of this concept was by Nicholson and Phillips [18] where both small cations and anions were employed. The ion of choice was released by iontophoresis from a micropipette located in the brain and the concentration measured as a function of time about 100 μm away using an ion-selective microelectrode. Subsequently, tetramethylammonium (TMA^+^) was used almost exclusively as the probe ion and the method became known as the Real Time Iontophoretic (RTI) method or TMA method. The technique was refined to allow for a small amount of loss of TMA^+^ [19] and also to permit ions to be delivered by pressure injection from a micropipette (the Real Time Pressure or RTP method). The basic equation for the RTI method, omitting terms for loss, is:

2

where *C*(*r,t*) is the concentration at radial location *r* from the source micropipette and *Q* is the source strength as a function of iontophoresis current and source transport number. This equation also only describes the rising phase of the curve while the current is applied (Figure 2A,B) – see Nicholson and Phillips [18], Nicholson [19] or Syková and Nicholson [20] for more detail. For the RTP method the basic equation, again omitting loss, is:
3

where a volume *U* of molecules at concentration *C*_f_ is released instantaneously from the micropipette. See Nicholson [21] and Syková and Nicholson [20] for more detail.

The RTI method has been used extensively by the Nicholson laboratory in New York with emphasis on the normal brain and by Eva Syková and colleagues in Prague, where many pathophysiological states have been investigated (for a comprehensive review see Syková and Nicholson [20]). The RTI-TMA method has amply confirmed the earlier studies by Fenstermacher and colleagues using sucrose or inulin; however, the RTI method has permitted studies with much higher spatial and temporal resolution so that, for example, anisotropic [22] and inhomogeneous properties [23] could be determined and the behavior of the ECS quantified in ischemic tissue (Figure 2B; [24, 25]).

The main limitation of the RTI and RTP methods is that the sensor is an ion-selective microelectrode so the method is constrained to a few select substances of low molecular weight (MW). It is thus best thought of as a probe of the structure of the ECS, as encapsulated in *α* and *λ*, although the method has been used to explore the interaction of Ca^2+^ with the extracellular matrix [26]. To extend the point-source paradigm to a wider variety of molecules, Nicholson and Tao introduced the Integrative Optical Imaging (IOI) method [27]. In this method, a fluorescent macromolecule was released from a micropipette by a brief pulse of pressure and the resulting cloud of diffusing molecules was imaged with a standard epifluorescence microscope. At a sequence of times, the solution to the diffusion equation as a function of distance (a Gaussian curve, see Eq. (3) as described for the RTP method) was fitted to fluorescence intensity and the tortuosity measured (Figure 2A). The method is not able to measure volume fraction because, unlike the iontophoretic method, the number of ejected molecules cannot be precisely controlled. The measurement of *λ* for a variety of molecules, including dextrans and albumins, has proved to be interesting and shown that *λ* increases with molecular weight rising from a typical value of 1.6 for TMA^+^ (measured with RTI) to greater than 2 for 70,000 MW dextran or 66,000 MW bovine serum albumin [20]. The method has even permitted the width of the ECS to be estimated as between 40–60 nm, using quantum dots [28]. The IOI approach has also revealed how proteins like lactoferrin interact with the heparan sulfate component of the extracellular matrix [29].

Diffusion studies are now a valuable adjunct in multimodal studies of function. For example, the RTI method has revealed that the ECS expands during sleep to facilitate the removal of waste products [14] and most recently both the RTI and IOI methods have helped elucidate the role of the extracellular matrix component hyaluronan and the ECS in epileptic seizures [30].

### Quantitation and modeling of diffusion in the ECS

All the diffusion studies of Fenstermacher and colleagues have relied on implicit models of diffusion in ECS. As noted above, the way the experiments were conducted led to diffusion in a single axis normal to the brain surface that was perfused with radiotracer. This mandated a one-dimensional solution to the diffusion equation for a slab of tissue subject to a constant concentration at one surface. This solution took the form of a complementary error function (Eq. (1)) and Fenstermacher’s longtime colleague, the late Clifford Patlak, a noted mathematician, devised special graph paper, which was based on the inverse of this function, to facilitate the analysis of early experiments. Deviations from the expected straight-line solution indicated that significant loss of the diffusing molecule from the ECS was taking place, through intracellular accumulation for example [15]. Another source of loss might occur across the BBB and this was modeled in the paper by Patlak and Fenstermacher [10]. It is worth noting the many important contributions of Patlak to the quantitative interpretation of diffusion in the brain; a study of Ca^2+^ diffusion in normal and thick slices [31] probably represents the most sophisticated analysis of radiotracers to date and may be compared with the later study using the RTP method by Hrabětová *et al*. [26].

The point-source paradigm also centers on a one-dimensional solution to the diffusion equation, but in this case the spherical symmetry of the problem dictates that the solution is carried out in a spherical coordinate system. In this approach, the diffusion is not driven by a constant concentration boundary condition, but either by a constant flux point source (RTI) or a small bolus (less than 1 nL) of injected substance (RTP and IOI). The very small source enables diffusion characteristics to be determined in a volume of tissue with a characteristic length of ~100 μm and in periods of 30–90 s.

Analysis of studies of diffusion of molecules in the ECS has thrown light on this most inaccessible brain compartment. Today the interpretation of the data often involves computer models. For example it was thought for a long time that the typical value of *λ* = 1.5–1.6 for ‘point’ molecules could be accounted for by the extra distance a diffusing molecule had to travel as it navigated around cellular profiles. Detailed Monte Carlo modeling using the MCell program (http://www.mcell.org) showed, however, that the increase in tortuosity attributable to this mechanism was generally less than or equal to 1.225 [32, 33]. Experiments by Hrabětová and Nicholson [24] and further application of Monte Carlo simulation showed that one way to increase the tortuosity was to incorporate so-called dead-spaces into the description of the ECS to elevate the tortuosity to the measured values. Imagine a model brain region consisting of cubic cells with a cubic cavity cut out from one corner (Figure 2C, inset) to provide a dead space when all the modified cubes are packed together. When the cubes are packed they remain separated by extracellular space with total extracellular volume fraction, *α*, of which the volume fraction of the cavity alone is *α*_c_, then, as shown by Tao *et al*. [34]:
4

where *β* = 3 for the geometry depicted in Figure 2C. As already noted ([26, 29]), for some molecules, interaction with the extracellular matrix may also increase tortuosity and this too can be modeled [35].

### The pathophysiology of the CSF

The diffusion of molecules within the ECS was not the only aspect of brain fluid physiology on which Fenstermacher focused his research. Indeed, he also had a keen interest in the CSF. It is quite likely that some of Fenstermacher’s early work related to CSF function was inspired by his interactions with neurosurgeons Thomas Milhorat and Mary Hammock, whose primary interest was in the pathophysiology of hydrocephalus. These were the late 1960’s and early 1970’s, and at that time ventriculocisternal perfusion was the technique of choice to measure the rate of CSF formation in experimental animals. Ventriculolumbar perfusion, a modification of the ventriculocisternal perfusion method, was sometimes used to measure the rate of CSF production in humans. Using this technique, Fenstermacher, Milhorat, Hammock, and others have shown that in a two-year-old child diagnosed with choroid plexus papilloma in one of the lateral cerebral ventricles, the removal of the tumor resulted in a considerable reduction in the rate of CSF formation [36]. Initially, inulin was employed as a non-diffusible marker for ventriculocisternal perfusion; however, concerns have arisen that the CSF formation rate could have been overestimated because of some diffusional loss of inulin from the perfusate into the brain tissue. Fenstermacher together with Robert Curran and other colleagues approached this problem by simultaneously using ^3^H- or ^14^C-labeled inulin and ^131^I-albumin as non-diffusible markers [37]. These studies clearly demonstrated an uptake of inulin by brain tissue surrounding the cerebral ventricles and emphasized the importance of using the high molecular weight markers, such as albumin, in the ventriculocisternal perfusion experiments. The group then went on to use Blue Dextran 2000, with a MW of 2 × 10^6^ Da, as a non-diffusible marker [38]. Since then Blue Dextran 2000 has been predominantly chosen as a marker to measure the rate of CSF formation with the ventriculocisternal perfusion technique.

Fenstermacher has also been interested in extrachoroidal sources of CSF. Earlier studies had suggested that the choroid plexus might not be the only source of CSF. Together with Milhorat, Hammock, and others, Fenstermacher demonstrated that in choroid plexectomized rhesus monkeys, the production of CSF was reduced by 33–40% [39]. While these experiments supported the extrachoroidal production of CSF, it is important to note that plexectomy is a highly invasive procedure, which by itself can have a significant adverse effect on brain function. Therefore, in view of our current understanding of brain physiology, these results should be interpreted with caution. In a later study [38], using the ventriculolumbar perfusion technique in rhesus monkeys, Fenstermacher together with Warren Lux showed that CSF is not produced within the spinal subarachnoid space. Working together with Victor Levin and other colleagues on a monkey model of acute obstructive hydrocephalus [40, 41], Fenstermacher has also shown that non-communicating hydrocephalus is associated with a significant expansion of the ECS in the periventricular white matter, whereas the production of CSF is unchanged under these pathological conditions.

The collaboration with Daniel Gomez, Gordon Potts, and others resulted in important insights into the CSF absorption pathways, [42, 43]. Using horseradish peroxidase as a tracer, the group demonstrated the involvement of the lymphatic system in bulk CSF absorption. This provided evidence for the complexity of CSF absorption process and allowed later on for a better understanding of the pathophysiology of neuroinflammatory diseases.

### The anatomy and physiology of CSF compartments in the brain

**Figure 3:**
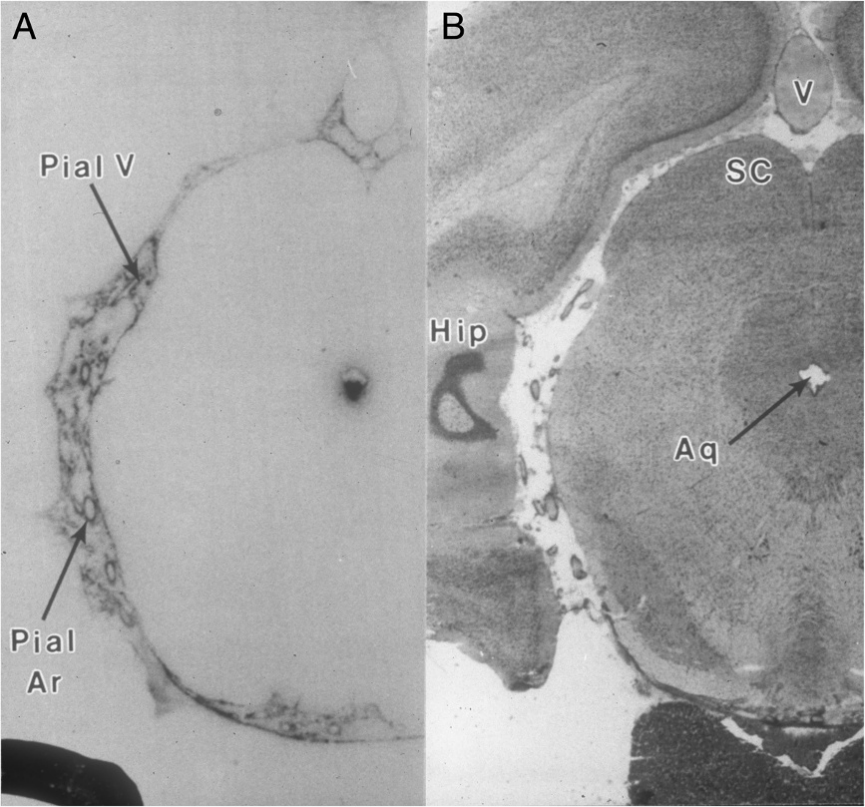
**Distribution of CSF-borne substances within the brain. A**. The autoradiogram shows the CSF-brain distribution of ^125^I-labeled soluble amyloid β-peptide (I-sAβ) 30 min after unilateral intracerebroventricular administration. The radioactive material was mostly visible around pial vessels (arteries) within the interpeduncular, ambient and quadrigeminal cisterns. The appreciable amounts were also present in the lateral recesses of the fourth ventricle (not shown) and along the ventral wall of the aqueduct. **B**. The histological microphotograph of adjacent coronal section of the brain with attached pituitary gland (Hip–hippocampus; SC - superior colliculus; V–large central vein; Pial V–small pial veins; Pial Ar–pial arteries; Aq–aqueduct, (adapted with permission from [48]).

His long-lasting interest in CSF physiology prompted Fenstermacher to investigate the peculiar tracer diffusion phenomena that were often observed in autoradiographic studies in the vicinity of fluid-filled spaces in the brain, the areas generally discarded from the analysis in most studies. He undertook an effort to track the flow of CSF and the fate of CSF-borne substances. His group combined cerebroventricular infusion of polar tracers that did not cause any disturbance in CSF flow with quantitative autoradiography (QAR) that involved brain tissue sampling from the frozen head to keep all fluids in place. This allowed the group to analyze the complex CSF circulatory system of cisternal and subarachnoid compartments, including the routes of CSF flow through the cisterns of the velum interpositum and anterior medullary velum. Together with his colleagues, Fenstermacher generated the tracer concentration-time profiles for multiple compartments and described a dual physiological role of glia limitans both in restricting the rate of tracer diffusion into neuropil and in acting as a reservoir for solutes carried by the CSF [44]. He also delineated the movement of CSF-borne tracers within the arterial/arteriolar perivascular spaces [44], supporting the pioneering work of Helen Cserr on the drainage of brain interstitial fluid along the Virchow-Robin perivascular spaces [45, 46]. In particular, Fenstermacher showed that the movement of solutes occurs from ventricle-to-interstitium-to-perivascular space. He also provided the tracer-based functional evidence for differences in perivascular anatomical organization and local perivascular flow along the cerebral arterioles and venules that had previously been discovered by Roy Weller’s group [47]. This paved the way to further studies of the role of brain fluids in the elimination of potentially deleterious endogenous metabolites. Applying the previously used techniques to study the clearance of CSF-borne substances from the brain, Fenstermacher together with the Blas Frangione group from New York University showed a biphasic CSF clearance rate for amyloid-β peptide_1–40_ [48]. While moving along the ventricular system, this peptide was initially rapidly cleared, which was proposed to occur across either the BBB or the BCSFB in the choroid plexus. This hypothesis has later been tested by his and other laboratories [49, 50]. After an initial rapid phase of clearance from the CSF, the elimination of amyloid-β peptide_1–40_ was slower when this peptide was moving through the subarachnoid/cisternal spaces to be eventually retained around pial arteries and arterioles (Figure 3). More recently, the role of the peculiar anatomical organization of CSF compartments and interconnected perivascular spaces in the clearance of amyloid-β peptide has been revisited. In these studies, a new brain imaging methodology was used and new hypotheses concerning the pathophysiology of Alzheimer’s disease were proposed [13, 14]. The detailed description of CSF flow pathways also had an impact on the research on neuroinflammation. In fact, the choroid plexus together with the subarachnoid and cisternal fluid spaces described by Fenstermacher and his colleagues are now considered to be key players in both normal neuroimmune surveillance and pathological neuroinflammation [51–56].

Studies on the clearance of CSF-borne substances from the brain were continued in Fenstermacher’s laboratory at the Henry Ford Hospital in Detroit, Michigan. Using ^125^I-insulin-like growth factor-1 (IGF1) as a tracer for this growth factor, the group demonstrated in a rat model that the entry of IGF1 into normal brain parenchyma after its intracerebroventricular administration was limited by a rapid clearance from CSF and brain, and by slow diffusion into the periventricular brain tissue [57]. Apart from these observations, two more novel facts were reported in this study. The first one was the deceptively similar-shaped temporal plasma profiles of radioactivity following both properly performed intracerebroventricular and erroneous intraparenchymal injections of the tracer. The second was the construction by Patlak of an ‘emergence function’ that could be used to estimate the rate of appearance of the tracer in the venous system.

Another interesting work concerning the elimination of CSF-borne substances, particularly drugs, was done in collaboration with David Smith and Richard Keep from the University of Michigan, Ann Arbor [58]. The purpose of these studies was to define the kinetics of CSF clearance, choroid plexus uptake, and parenchymal penetration in various brain regions of substrates for an oligopeptide transporter PEPT2 (SLC15A2) following their intracerebroventricular administration. PEPT2 is a high-affinity, low-capacity carrier belonging to the family of proton-coupled oligopeptide transporters. It is expressed at the apical surface of the choroid plexus epithelium as well as in astrocytes (newborn rats) and neurons (newborn and adult rats). Residing at the BCSFB, PEPT2 plays a significant role in limiting the brain exposure to CSF-borne peptides and peptide-like drugs by transporting these substances out across the BCSFB. These studies demonstrated that the deletion of the *Pept2* gene in mice significantly modifies the spatial distribution of glycylsarcosine (GlySar) and cefadroxil (and presumably other peptides and peptide-like drugs) in brain at the CSF-choroid plexus interface, ependyma, and the septum. The penetration of the periventricular brain tissue by ^14^C-GlySar in PEPT2-null mice significantly differed from that observed in wild-type animals, providing evidence for the importance of PEPT2 in parenchymal drug distribution. This was the first study to demonstrate an uptake and retention of a PEPT2 substrate in the choroid plexus tissue in all four cerebral ventricles using QAR. Thus, combining the gene-deletion technology with high spatial resolution QAR proved to be a valuable approach for studying the role of brain barriers in the regional distribution of drugs in the brain.

### The physiology of the BBB

From early on in his scientific career, Joseph Fenstermacher had a significant interest in physiology of the BBB. In his first study published together with John Johnson [59], he assessed basic properties of the BBB, such as the filtration and reflection coefficients, for water and several solutes, including glucose, sucrose, and urea. Later on Fenstermacher teamed up with Patlak to measure the influx rates across the BBB for water and various solutes using the ventriculocisternal perfusion technique in dogs [10]. It seems that for Fenstermacher it was critically important to know all the advantages and disadvantages of the techniques he was using. This is reflected by his interest in theoretical analyses, like for example the analysis of the experimental conditions for the measurement of the blood-to-brain transfer constants that was done together with Patlak and Ronald Blasberg [60].

Fenstermacher also spent some summers working at the Mount Desert Island Biological Laboratory located in charming Salisbury Cove, Maine, where he met Helen Cserr. As mentioned above, Cserr was a pioneer in research on bulk flow of interstitial fluid in the brain. Later on she also investigated the pathophysiological mechanisms underlying the brain immune response. Their collaboration resulted in publications analyzing the properties of brain barriers in sharks and other marine species [61, 62]. The research performed in the same laboratory together with Andreas Roomet provided data on CSF production and absorption in sharks [63].

### The opening of the BBB in acute ischemic stroke

Previous studies had indicated that the BBB damage after stroke not only varies in regard to its spatial distribution, but also in its magnitude within a given brain region [64]. Such information was indirectly inferred from the differential distribution of radiolabeled tracers with two different molecular sizes, such as sucrose and inulin [65]. Our previous data suggested that the gadolinium-diethylenetriaminepentaacetic acid (Gd-DTPA) enhancing regions observed immediately after reperfusion developed hemorrhagic transformation (HT) at 24 h post-stroke [66]. This prompted Fenstermacher and his colleagues to postulate that an acute extravasation of blood-borne substances must be limited by the size of the opening of the BBB. This hypothesis was investigated in a series of innovative experiments. In the first set of experiments, the transvascular movement of plasma and red blood cells (RBC) in acute stroke and 24 h after reperfusion was studied. Classical QAR tracers available for such studies were radioiodinated serum albumin (RISA) and ^55^Fe-labeled RBC [67]. Instead, the Fenstermacher group used a technique previously employed in tumor blood flow experiments [68] with Evans blue, a plasma marker (MW of ~66 kDa after binding to plasma albumin), administered together with fluorescein isothiocyanate (FITC)-labeled autologous RBCs. The data showed that at 3 h after reperfusion, Evans blue leaked, but the RBCs remained intravascular, whereas at 24 h post-reperfusion both Evans blue and RBCs were found in the extravascular space [69], confirming the previous observations by Knight *et al*. [66]. The next question to address was the maximal size of blood-borne molecules that can extravasate acutely after ischemia. For these investigations, once again Evans blue bound to plasma albumin was chosen as the reference tracer, which was used together with a series of FITC-labeled dextrans of varied sizes. These studies demonstrated that FITC-dextran of 2 × 10^6^ Da remained intravascular acutely after reperfusion, suggesting a possible size limit for blood-borne substances penetrating the BBB after cerebral ischemia [70].

The demonstration of variable magnitude of BBB damage within the ischemic lesion was done later using the MRI technique and employing for the first time Gd-DTPA and Gd-DTPA-albumin, two contrast agents with a significant molecular size difference [71]. Gd-DTPA-albumin was also linked to Evans blue, enabling the fluorescent microscopic confirmation of the patterns of magnetic resonance (MR) distribution of Gd-DTPA-albumin. These data conclusively proved for the first time that the BBB damage varies within the ischemic lesion and that it could be imaged *in vivo*.

### Early studies of the endothelial barrier in brain tumors

Working at the National Cancer Institute (NCI) in Bethesda, Maryland, Fenstermacher together with Blasberg, Peter Molnar, and others, investigated the properties of the endothelial barrier in brain tumors. Using an experimental rat model, the group studied various brain tumors, including the avian sarcoma virus (ASV) [72] and ethylnitrosourea-induced tumors [73], as well as tumors produced by intracerebral injection of RT-9 rat gliosarcoma cells [74] or intracarotid delivery of Walker 256 rat carcinoma cells [75]. The ethylnitrosourea model was especially useful for these studies, as it produced diverse tumors, including ependymomas and a variety of glial tumors, such as gliomas, astrocytomas, oligodendrogliomas, and schwannomas [73]. A local unidirectional blood-to-tissue transfer rate constant for ^14^C–α-aminoisobutiric acid (AIB) – a low molecular weight tracer – was measured in these tumors using QAR. A significant variability in a unidirectional blood-to-tissue transfer rate within tumors was shown, with the values of influx rate frequently depending on the size of the tumor and its location. However, the magnitude of the transfer rate was generally unrelated to specific histological features of the tumors. These studies had important translational implications for targeting brain tumors in humans.

### Research on regional CBF and its regulation

In 1983 Fenstermacher moved from the NCI to the State University of New York at Stony Brook to resume his research on CBF and the coupling of local blood flow to metabolism in the brain. He was soon joined there by Clifford Patlak. Fenstermacher, with his research team members Ling Wei, Daniel Bereczki, Tada Otsuka, Jiann-Ling Chen, Atsushi Tajima, and Franz-Josef Hans, refined the techniques for measuring local CBF, local cerebral glucose utilization, and the regional capillary permeability coefficient and the surface area (PS) products for various compounds. The group combined various protocols of intra-arterial administration of tracers/indicators (e.g., ramp infusion) in both awake and anesthetized rats with either tissue sampling from discrete brain areas or QAR. The data obtained in these experiments were analyzed using mathematical models developed by Patlak. This work generated a vast amount of data on regional differences in blood volume, hematocrit, CBF, and PS products for tracers for lipid-mediated and carrier-facilitated transport across the BBB for 40 distinct brain regions [67, 76–80]. These results now constitute the reference data that are invaluable in interpreting the regional differences in various experimental settings in the field of neurobiology. This methodology was employed to study animal models related to toxicology (nicotine [81, 82]), the effects of anesthesia (pentobarbital [67, 83]), physiological stress, such as hypoxia [77, 79] and hypercapnia [76, 78], and in some diseases, such as hypertension [80]).

A good example of research conducted at Stony Brook was investigation into the physiological mechanisms regulating CBF. In the 1990’s a generally accepted theory was that an increase in CBF observed in response to hypercapnia or hypoxia resulted from the capillary recruitment rather than an increase in blood flow through already perfused capillaries. Fenstermacher and his colleagues decided to take a close look at this controversial issue. Using ^125^I-labeled serum albumin and ^55^Fe-labeled RBC to estimate the blood volume in parenchymal microvessels, Bereczki *et al*. [78] demonstrated that while hypercapnia – one of the major factors regulating blood flow to the brain – caused a significant increase in CBF, this increase was predominantly associated with the rise in the velocity of flow of RBC and plasma through already perfused capillaries. The data provided evidence for only modest capillary recruitment in response to hypercapnia. These observations were confirmed by a later study [76] in which the PS products for ^14^C-iodoantipyrine (IAP) and ^14^C-3-*O*-methyl-D-glucose (3OMG) were assessed. The group has also investigated the mechanisms underlying the hypoxia-dependent regulation of CBF. Being another important factor controlling CBF, hypoxia too has been shown to increase CBF mainly by increasing the velocity of perfusion of brain microvessels [79]. The assessment of PS products for IAP and 3OMG performed in the subsequent study [77] also provided evidence to disprove the hypothesis that an increase in CBF in response to hypoxia is associated with capillary recruitment.

In addition to brain physiology, Fenstermacher had a deep interest in the intricacies of research techniques used to measure blood flow to the brain. In fact, Fenstermacher’s early involvement in the field of CBF began with his contribution to theoretical work on CBF. Together with Patlak, Louis Sokoloff, and other collaborators [84], he described experimental limitations of IAP, a diffusible indicator most commonly used to measure local CBF by QAR. He then went on to evaluate the errors in the measurement of CBF by the indicator fractionation and tissue equilibration methods [85]. This theoretical work was done together with Patlak and Blasberg.

As described above, Fenstermacher collaborated with Blasberg, Molnar, and others to better understand the pathophysiology of brain tumors. They not only investigated the properties of the endothelial barrier in brain tumors, but also were interested in how these tumors are perfused. The group studied the ASV- [86] and ethylnitrosourea-induced tumors [87], as well as tumors produced by RT-9 rat gliosarcoma cells [88] and Walker 256 rat carcinoma cells [89]. The ^14^C-IAP-based QAR was used to assess local CBF in these tumors. Similar to the measurements of endothelial permeability to AIB in brain tumors, these studies have demonstrated remarkable heterogeneity in perfusion of the tumors, with the levels of blood flow being frequently unrelated to histological findings. In addition to characterizing the blood flow patterns in brain tumors, these studies had important therapeutic implications. The concurrent measurement of blood flow and capillary permeability to AIB in ASV-induced tumors demonstrated that the capillary permeability and surface area, and not tissue perfusion, determine the blood-to-tissue influx of blood-borne materials in brain tumors [90].

In later studies, Fenstermacher together with Paul Gross and other colleagues has focused on analyzing functional and structural differences in capillary endothelium between various brain regions. The group was particularly interested in circumventricular organs (CVOs), such as the subfornical organ and median eminence, because of their anatomical location and distinct morphological features. While the rate of capillary blood flow to CVOs was found to be similar to that observed in brain grey matter, Fenstermacher and his collaborators showed that the PS product for AIB in CVO capillaries is 300 times greater than that measured in capillaries constituting the BBB [91]. These findings were not only consistent with morphological features of endothelial cells forming the capillaries in CVOs, but also supported the putative roles of CVOs in diverse physiological regulatory processes.

### Concurrent application of QAR and terminal tracer techniques with MRI and other imaging modalities

The last seventeen years of his career Joseph Fenstermacher spent working at the Henry Ford Hospital in Detroit, Michigan. A significant part of his work there involved employing MRI to investigate cerebrovascular pathology in animal models of ischemic stroke and brain tumors. Several other studies investigating the drug distribution in the prostate gland and the theoretical aspects of water proton tracking, as well as the analysis of histopathological changes in ischemic brain underlying MRI signal changes were also done there. In all these studies, Fenstermacher and his team used QAR and histopathological techniques as a gold standard to validate MR imaging signals for assessing CBF, BBB permeability, ECS, etc. This approach aided in precise localization and quantification of cerebrovascular pathology by MRI. Several ‘Aha’ moments were a part of the scientific discussions. Over the decades Fenstermacher and his colleagues painstakingly perfected techniques for measuring CBF, blood-to-brain transport kinetics, ECS, fluid bulk flow, diffusion, convection, etc., forming the basis for imaging applications in clinical practice.

### Stroke studies: CBF measurements using MRI

One of the first major undertakings was to compare two MRI arterial spin tagging (AST) methods for estimating CBF with the classic IAP-based QAR technique [92]. A rat ischemic stroke model with suture occlusion of the middle cerebral artery for 2 h was employed in these studies. This model had the advantage of producing an extended range of CBF rates for testing the sensitivity of the measurements and a normal contralateral side for comparison in the same rat. Two MRI approaches were used: spin echo (SE) and variable tip angle gradient echo (VTA-GE) readouts. Despite the widespread use of AST-MRI techniques to estimate CBF, very few studies were done till then to compare the MRI data with established terminal tracer studies. For instance, single-coil AST has been used in the laboratory as an imaging technique for assessing the state of cerebral perfusion in a variety of rat models of ischemia. Questions have been raised regarding whether it is advisable to eliminate signal from vascular spins, whether the technique produces signal linear to flow, and under what conditions the technique might produce an unbiased estimate of flow. It was in this framework that the group examined the operating characteristics of the AST-MRI measurement across the wide range of flows. For the AST studies, both SE and VTA-GE readings were used to assay tissue magnetization. The aim of this study was to determine whether there is a correlation between CBF estimates produced by the two AST-MRI methods; whether the CBF rates measured by either or both of the AST-MRI methods are concordant with those determined by IAP-QAR; and finally whether the AST-MRI methods yield robust estimates of CBF.

Nine brain regions were chosen as regions of interest (ROIs) for CBF assessment: preoptic area, caudate-putamen, globus pallidus, stria terminalis, piriform cortex, insular cortex, parietal cortex, hindlimb and forelimb cortex, frontal and cingulate cortex. The QAR data were acquired in two ways: 1) Traditional field-by-field method in which each ROI was outlined on a digitized image of the X-ray film and a standard curve for radioactivity *vs*. optical density was generated from ^14^C standards exposed along with brain sections. Since the imaged MRI slices were 2 mm thick, values across 5 QAR images (each from 400 μm thick brain sections) representing the virtual 2 mm MR slice were averaged to get a representative value. 2) Averaged method in which the QAR film was scanned and the images representing the 2 mm MRI slice were summed digitally. A template of the ROIs was then superimposed on this digital image for the acquisition of CBF values. The two sets of CBF values from these two methods were positively correlated. With this confirmation, Method (2) was used for further testing with MR data. The results showed that despite the 30–40-min time lag between MRI and QAR studies, both MR techniques were fairly well correlated with CBF rates calculated using IAP-QAR [93]. However, the QAR method produced slightly lower estimates that were better correlated with the VTA-GE values. Also, the correlation was better in the ischemic side than the contralateral side. The data also suggested that the SE-CBF estimates were better in high-flow rate ranges and those of VTA-GE in the low-flow range. Thus, these data indicated that using both these techniques in human imaging would lead to superior computation of CBF variations under disease conditions.

### The blood-to-brain influx of an MRI contrast agent in ischemic stroke and the Patlak plot

The MRI-QAR correlation of CBF in stroke demonstrated that the approach was feasible and valuable in confirming proton MRI data. This led to the next step of evaluation of BBB opening in stroke, its localization by contrast-enhanced (CE)-MRI, confirmation and quantification by an appropriate QAR technique. Such studies were essential since CE-MRI was employed to image BBB injury in stroke, brain tumors, and other CNS diseases; however, the quantification of BBB damage by MRI needed some more refinement. To this end, a rat model of transient cerebral ischemia resulting in hemorrhagic transformation at 24 h post-stroke was chosen [66]. A 3-step series of experiments was planned to accomplish the objectives. Methods were very similar to those adopted for CBF studies, with each MRI session immediately followed by a QAR experiment in the same rat and the data from QAR being a gold standard to confirm the MRI results. The MRI contrast agent used in all these studies was Gd-DTPA. Gadolinium analogs are the most commonly used clinical paramagnetic agents. The first QAR tracer chosen for comparison was ^14^C-sucrose. The distribution of ^14^C-sucrose in the brain had been previously studied by Fenstermacher and colleagues [7–10]. The MW for ^14^C-sucrose (302 Da) is close to that of Gd-DTPA (565 Da), and most importantly, both Gd-DTPA and sucrose do not have any known uptake mechanisms in the brain. Therefore, their diffusion within the ECS or their back efflux to the vasculature should result in identical distribution patterns enabling one-to-one comparisons.

Using the above described rat stroke model, CBF, diffusion weighted imaging, T_2_, T_1_ and magnetization transfer parameters were acquired during arterial occlusion and again after reperfusion. CE-MRI was performed once at about 2.5 h after initiating reperfusion with a Look-Locker T_1_-weighted imaging sequence. At the end of the MRI session, the rat was removed from the magnet and infused with a ^14^C-sucrose step-down protocol. Timed arterial blood samples were collected for constructing the time-concentration curve or arterial input function (AIF). Rats were sacrificed and brain tissue sections along with ^14^C standards were used to generate images for QAR. The blood-to-brain influx constant (K_i_) and the extravascular distribution volume (v_e_) were computed from ^14^C-sucrose AIF and the brain regional concentrations calculated from X-ray films. While the QAR calculations were straightforward, equivalent MRI calculations proved troublesome. The conventional convolution/deconvolution-based K_i_/K^trans^ estimates were inconsistent and not in agreement with QAR estimates. As suggested by James Ewing, one possible way to address for this discrepancy was that during the MRI acquisition, the sagittal sinus was imaged and therefore its blood contrast agent (CA) levels were temporally measured during the Look-Locker sequence along with tissue levels in terms of R_1_ (R_1_ = 1/T_1_). That was one of the many ‘Aha’ moments in the lab when Joe Fenstermacher said: “Jim, you are describing the Patlak plot” [94, 95]. Initially, for the construction of the Patlak plot, which described the blood-to-brain compartmental tracer distributions, rats were injected with the tracer and its AIF was measured. However, the rats had to be sacrificed at various durations of tracer circulation to assess the brain tracer levels by autoradiography. Brain entry after various circulation periods was then calculated and plotted with the slope of the linear part of the resultant curve representing the influx rate constant K_i_. Now the MRI estimates of both blood and brain concentrations of the tracer could be used to construct a Patlak plot. This was reported by Ewing *et al*. [96] and Fenstermacher *et al*. [97].

The Patlak plot was also used to investigate whether the Gd-DTPA method would allow the group to localize BBB openings. ^14^C-labeled AIB was chosen as a QAR tracer. AIB is the best existing tracer for the localization of BBB opening and the methodology of its use was well established [98]. A rat stroke model was employed in these studies along with CE-MRI, and the correlation of non-contrast-based MRI parameters, such as the magnetization transfer and T_1sat_, with BBB damage was reported in two publications by Knight *et al*. [99, 100]. These data suggested that the Gd-DTPA-based CE-MRI could represent a good indicator of the BBB damage in acute stroke. Changes in parameters, such as T_1sat_ and T_1_, may also be indicative of BBB damage due to their sensitivity to fluctuations of water across the BBB. However, it is important to note that the spatial resolving power of CE-MRI with a bolus CA injection is limited to large BBB lesions.

The third and final step in comparing both methodologies came with the use, for the first time, of identical tracers in MRI and QAR studies. Gd-DTPA was used for the CE-MRI studies, whereas its equivalent Gd-^14^C-DTPA was employed for QAR. At variance with the previous studies in which the MR data were analyzed by Patlak plots and compared to QAR data derived using the single time equation, both MR and QAR data from this series of experiments were analyzed by the single time equation [60]. These data were also compared to the values derived from the Patlak plot. This was done to ensure that not just the tracers, but also the analysis would be identical between the two techniques. Results showed that the regions of BBB opening were very similar on the MRI maps and the autoradiograms. The extravascular distribution volume was nearly identical for Gd-DTPA and Gd-^14^C-DTPA, and K_i_ was slightly, but not significantly, higher for Gd-DTPA *vs*. Gd-^14^C-DTPA. The K_i_ values were also well correlated. When the arterial concentration-time curve of Gd-DTPA was adjusted to match that of Gd-^14^C-DTPA, the two sets of K_i_ values were alike and statistically comparable with those previously obtained from a Patlak plot. These findings demonstrated that the CE-MRI technique accurately measures the Gd-DTPA concentration in blood and brain, and that the K_i_ estimates based on such data are good quantitative indicators of BBB injury [101].

Separate studies in which Gd-DTPA was either injected as a bolus or infused using the step-down infusion (SDI) method demonstrated that the SDI method results in superior signal-to-noise ratio with far brighter pixels in the enhancing/BBB-damaged regions [102]. This method was also advantageous in capturing the complete AIF curve more accurately than the bolus that missed the peak CA blood concentration. When analyzed further, it was discovered that missing the peak blood concentration led to an overestimation of plasma volume (v_p_) but did not seem to affect K_i_ calculations [94]. Another important finding from these experiments was that a population-averaged AIF could be used for estimating BBB kinetics if direct measurements were not possible in every study [94]. As will be discussed later, direct radiotracer-based AIF measurements for Gd-DTPA from these experiments were useful for estimations of tumor vascular permeability kinetics.

The superior contrast enhancement after SDI administration of Gd-DTPA suggested that this might be useful in a better demarcation of the BBB opening. This idea was tested by CE-MRI studies using again the ischemia-reperfusion rat model with SDI administration of Gd-DTPA in the magnet and Gd-^14^C-DTPA on the table for QAR. Identical patterns of CA blood levels were recorded in both procedures. The normalized plasma concentration-time integrals were identical for Gd-DTPA and Gd-^14^C-DTPA, indicating that the MRI protocol yielded reliable estimates of plasma Gd-DTPA levels [103]. In rats with BBB opening, 14 spatially similar regions of extravascular Gd-DTPA enhancement and Gd-^14^C-DTPA leakage, including one very small area, were observed. The terminal tissue-plasma ratios from QAR tended to be slightly higher than those from MRI in these regions, but the differences were not statistically significant. The MRI-derived K_i_ values for Gd-DTPA closely agreed and correlated well with those obtained for Gd-^14^C-DTPA. Apart from these confirmations, a salient feature of these studies was that compared to bolus injections, spatial resolving power of the SDI input was greater with BBB openings as small as 0.5 mm^3^ detected by CE-MRI [103]. Subsequent analysis also showed that unlike the bolus injection, the SDI protocol allows for the magnetic resonance contrast agent (MRCA) to spread beyond the ischemic core [104].

### Brain tumor Gadomer and Gd-BSA studies

Despite the often-observed vascular leakiness, primary brain tumor vascular pathology differs from that of the BBB leakage occurring in acute stroke. It is characterized by chronically dysregulated blood flow, angiogenesis with areas of differentiation and necrosis. In some tumors, the extreme leakiness of the vessels may result in a rapid clearance of small molecules, such as Gd-DTPA, leading to either under- or overestimation of permeability kinetics. Therefore, an experimental dendritic, macromolecular magnetic resonance contrast agent (MMCA) Gadomer-17 produced by Schering AG (Germany) was used in brain tumor studies to overcome these disadvantages. Gadomer-17 has a MW of 17 kDa with an effective size nearly that of plasma albumin. As its equivalent, another MMCA was prepared by linking bovine serum albumin (BSA) to Gd via the chelating agent DTPA. The resultant product had a MW of about 90 kDa, with Gd:BSA ratio of 14:1 (as opposed to Gd:DTPA ratio of 1:1 in the Gd-DTPA complex) [68]. The contrast enhancement patterns of Gadomer-17 and Gd-BSA were sequentially evaluated in a rat 9L gliosarcoma model. Following intravenous injection, the blood concentration of Gadomer-17 fell rapidly, whereas that of Gd-BSA was almost constant for the duration of imaging. The areas of enhancement of both MMCAs were comparable probably owing to their similar effective sizes. The spatial distribution of Gd-BSA also showed a good agreement with that of Evans blue-tagged albumin. Treatment with dexamethasone decreased Gd-BSA enhancement in the tumor, suggesting that it can be used to image brain tumors and their response to treatment [105].

As the final confirmation of these data, the classic concurrent MRI-QAR experiments were performed using the same tumor model. With Gd-BSA as the paramagnetic agent, RISA was employed for QAR. Look-Locker MRI estimates of T_1_ followed Gd-BSA blood (sagittal sinus) and tissue (ROI) concentration. QAR and MRI maps of K^trans^ were co-registered, an ROI that included the tumor and its surrounding brain tissue was selected, and the two estimates of K^trans^ from the ROI on QAR and MRI maps were compared by either mean per animal ROI or on pixel-by-pixel data using a generalized estimating equation. Good correlation was observed between end-point QAR values and MR parameters whether calculated as mean values per ROI or as pixel-by-pixel values within each ROI [106].

### Model selection paradigm

Vasculature within a solid tumor exhibits a range of leakiness and visualizing the enhancement patterns is not always reflective of these variations. Ability to segment and quantify such variations is important since they can serve as measures of loci of tumor aggressiveness, whereas changes in their patterns can represent biomarkers of response to treatment. Moreover, with the minimally invasive CE-MRI techniques, longitudinal monitoring of such features is possible. With its ability to act as a reliable marker for tumor vascular permeability, Gadomer-17 was selected as the MRCA of choice to image and segment tumor vascular functional variations.

The rat 9 L gliosarcoma model was used in the studies designed to describe the operating characteristics of MRI estimates of tumor vascular permeability. An extended Patlak plot model was employed to establish regions with: (1) no vascular leakage (Model 1 with just plasma volume, v_p_); (2) leakage with no backflux (Model 2, with v_p_ and forward volume transfer constant K^trans^); and (3) leakage along with measurable back flux (v_p_, K^trans^ and k_ep_). An objective F statistic was used to segment these regions. The prevailing vascular permeability conditions determined which model satisfies the F test requirement best, thus eliminating selection bias. It should be noted, however, that in not just brain tumor models, but also in other models of neurovascular disease, there is almost certainly a broad range of microvascular permeabilities among and within lesions. This range is the result of the disease processes and is essential for disease and treatment evaluations. To this end, the simplicity of calculating influx parameters by the extended Patlak plots and the objective application of F statistic to segment the different regions were established by these observations [107].

While it was apparent that serial MR investigations are possible in rat brain tumor models to evaluate treatment effects, the model selection paradigm required further confirmation to establish its sensitivity to mirror acute treatment effects. The often-used glucosteroid dexamethasone was chosen for this purpose due to its known fast action in decreasing peritumoral edema. Moreover, previous studies by Fenstermacher’s group had established the basic vascular constants and effects of dexamethasone using QAR [108]. Thus, the gold standard values were readily available for the comparison. Gadomer-17 was used for CE-MRI and a single, high dose of dexamethasone (8 mg/kg) was used to achieve quick therapeutic effects. The dose of dexamethasone was chosen based on previous work on animal brain tumor models in which between 3 and 30 mg/kg of dexamethasone was shown to be effective in lowering the interstitial tumor pressures and reducing edema. After the first MRI estimate of permeability (‘Test’), dexamethasone was administered intravenously. A second permeability study (‘Retest’) was performed 90 min after the administration of dexamethasone. Results demonstrated that model selection effectively captured the effect of dexamethasone on tumor vasculature [109]. Using significantly fewer rats the data also confirmed an earlier work of Fenstermacher and his colleagues [108].

### Logan plot and measuring tumor response to therapy

After establishing the MRI operating characteristics and the model selection paradigm for tumor evaluation, investigations were extended to examine their potential to serve as biomarkers for the efficacy of interventions such as the use of anti-angiogenic and anti-vascular agents, drugs for tumor cell kill, and radiotherapy. An immunocompromised athymic nude rat model of U251 glioblastoma along with a dual echo-gradient echo dynamic contrast-enhanced (DCE)-MRI were used for these studies. Their purpose was to measure the increased cell packing density in tumors compared to normal brain using DCE-MRI. Apparent diffusion coefficient (ADC) of water was already reported to correlate with cell density. However, the group felt that it could develop a DCE-MRI measure of interstitial volume fraction (also known as porosity), changes in which may reflect alterations in cell density and correlate that to histological measurements of actual cell density in the ROIs. If achieved, this could then be used to non-invasively measure the effects of tumor cell kill agents. Thus, the objective of this study was to test the hypothesis that tumor cellularity and DCE-MRI-derived interstitial volume fraction (v_e_) and/or distribution volume (V_D_) were correlated in experimental cerebral tumors. Using a Standard Model analysis [110] and the Logan graphical plot [111], DCE-MRI image sets during and after the injection of a clinically used Gd contrast agent Magnevist, were employed to estimate the parameters v_p_, K^trans^, v_e_, and V_D_. This was most likely the first attempt to apply the Logan graphical plot to DCE-MRI data. Unlike the Patlak plot and the Standard Model, the Logan plot uses the later part of the uptake curve (when the response function either does not change with the input function or is at a nearly steady state) for calculations. As an additional confirmation, the cell density data were also compared with ADC values from these experiments. The results demonstrated that the Logan plot-estimated V_D_ correlated with the Standard Model’s v_p_ + v_e_ [112]. In addition, the parameters v_e_ and V_D_ significantly and negatively correlated with tumor cellularity. A significant correlation of cell density with ADC values was also observed, although this relationship was not as strong as those with DCE-MRI measures [112].

An interesting feature of these studies was the adaptation of the AIF from the previous radiotracer experiments. An accurate determination of the AIF is essential for precise calculation of permeability parameters. Because of the difficulty in directly estimating the AIF from the MRI data, a radiotracer assay-based input function obtained from the previous investigation [94] was used in the dynamic MRI studies. This demonstrated that, starting at about two minutes after injection of CA, the radiotracer and MRI measures of blood concentrations of CA track each other very well. It was assumed for this adaptation that: (1) no vascular leakage of CA occurred in the contralateral striatum, and (2) the plasma volume of the caudate putamen was 1%. The integrated area of the radiotracer AIF was scaled so that its area was 100 times that of the integrated area of the average value of ΔR_1_(t) in the caudate putamen of the opposite hemisphere.

Using the criteria established by such investigations, the acute effects of cilengitide, an anti-angiogenic agent, on tumor vascular permeability kinetics were measured. These data showed that cilengitde had a vascular normalizing effect at about 8 h after administration. This finding coincided with previous reports of increased treatment efficacy when radiotherapy followed cilengitde after 8 h [113]. The Logan plot was subsequently used to measure the peritumoral fluid flow, which is known to lead to brain swelling and edema that can be fatal. Pathophysiologically, its severity may also represent increased tumor interstitial fluid pressure (TIFP), a factor that impedes tumor drug penetration and decreases treatment efficacy. However, attempts to measure V_D_ beyond the peritumoral rim always resulted in a negative ‘y’ intercept of the Patlak curves. In previous experiments on vascular permeability the ‘y’ intercept was positive and was considered to represent a measure of plasma distribution volume v_p_ of Gd-affected protons. The negative values led to several discussions to resolve the problem and in another ‘Aha’ moment, Fenstermacher and James Ewing realized that it was due to the fact that the driving force beyond the tumor rim was interstitial and not intravascular. The Gd-based Magnevist in the peritumoral region was being carried with the edema fluid toward normal brain. This suggestion once again has led to extensive data analysis and formulation of hypotheses about the tumor rim and the presumably normal, adjacent brain and their probable roles in tumor perfusion and TIFP.

### Other studies

Along with the major research advances in cerebral ischemia and brain tumor pathophysiology, Fenstermacher and his collaborators pursued many other small projects. Together with Jae Ho Kim, Svend Freytag and Steve Brown from Radiation Oncology at the Henry Ford Hospital, Fenstermacher worked on optimizing adenovirus-mediated suicide gene therapy for prostate cancer. They developed a method based on the human sodium iodide symporter (hNIS) that allowed for noninvasive monitoring of adenoviral vectors and quantification of gene expression. High-resolution autoradiographs of prostate sections coupled with a 3D reconstruction of gene expression were generated, demonstrating that the magnitude and volume of gene expression could be quantified [114]. The method demonstrated submillimeter resolution allowing for precise measurements of gene expression magnitude and volume in vivo [115]. Clinical trials on gene therapy for prostate cancer followed these publications.

New animal models of ischemic stroke were also developed as a result of Fenstermacher’s other collaborative projects. Together with Ling Wei, Dennis Choi and Chung Hsu, he investigated how the severity of stroke varies among different mouse strains [116]. In addition, a new technically challenging mini-stroke model was developed in rat with Ling Wei and Thomas Woolsey [117]. This model showed an exquisite structural and functional association with barrel cortex and provided a technique for functional testing of stroke treatments. A rat model of intracerebral focal stroke resulting from the photothrombotic lesion was also developed together with Toshihiko Kuroiwa, Guohua Xi, Ya Hua and Richard Keep [118, 119]. This model exhibited a clearly defined ischemic boundary along with BBB disruption. Although it was initially produced in the striatum, a small, focal lesion could be reproduced in any given brain region by changing the placement of the fiber optic tip.

Aside from many experimental studies, Fenstermacher also co-authored several theoretical papers. One of his most important accomplishments in this area was to enhance our understanding of the behavior of blood-borne water protons under a magnetic field. This provided the basis for AST measurements of CBF. In AST measurements, the protons as they flow along the carotid arteries are subjected to a magnetic field and their reorientation as they pass beyond the field generates the signals for the measurement of flow. Discussions on this topic led to publications [120–123], which in turn formed the foundation for the papers on multiparametric and DCE-MRI measurements. A similar approach was later followed in the development of a neural network method for semi-automated calculations for the objective assessment of image data sets [123–125].

The distribution of water in brain always occupied Fenstermacher’s thoughts due to its crucial role in health and disease and its significance as the basis for proton MRI. Fundamental studies in stroke on ADC changes and their relationship with simultaneous astrocyte swelling and neuronal shrinking to explain such changes in terms of cellular alterations were conducted in collaboration with Marc Fisher, Kai-Feng Liu and the late Julio Garcia [126–129].

## Conclusions

The various scientific accomplishments of Joseph Fenstermacher discussed in this review were not necessarily done in chronological order, but, rather, were often performed simultaneously, frequently influencing each other. The early works of Fenstermacher and colleagues on CBF, ECS, and the distributions of water and radiotracers across the BBB evolved seamlessly into the use of modern technologies, such as MRI and CE-MRI. Techniques were borrowed from one application to develop the tools for another project. Examples include, but are not limited to, FITC-labeled RBC used both for tumor blood flow studies and to estimate temporal evolution of BBB damage in acute stroke, the MRI-CAs utilized both in stroke and tumor studies, and the list goes on.

In the modern and fast developing world of science, when researchers frequently specialize in a unique and narrow discipline, scientists like Joseph Fenstermacher are unusual. He represents a rapidly diminishing generation with a deep understanding of the principles of physiology. Nowadays, many consider this discipline of biomedical science as rather archaic and unattractive. However, when a holistic approach to the problem, such as systems biology, is needed and the understanding of the basics of physiology is lacking, the use of even the most advanced and sophisticated technologies to solve the problem may not provide the correct answers. The quest for a better insight into physiology of fluids and barriers of the brain championed by Joseph Fenstermacher laid the groundwork for many of us to build upon. Although he retired in 2012 from the Henry Ford Hospital, he continues to write manuscripts and consult on grant applications, and stays in close touch with his colleagues. Jean-François Ghersi-Egea and Tavarekere Nagaraja, the contributors to this review, had the privilege to train with Fenstermacher at different stages of his career.

## Abbreviations

3OMG: 3-*O*-methyl-D-glucose
ADC: Apparent diffusion coefficient
AIB: α-aminoisobutiric acid
AST: Arterial spin tagging
ASV: Avian sarcoma virus
BBB: Blood–brain barrier
BCSFB: Blood-cerebrospinal fluid barrier
BSA: Bovine serum albumin
CA: Contrast agent
CBF: Cerebral blood flow
CE-MRI: Contrast-enhanced magnetic resonance imaging
CSF: Cerebrospinal fluid
CVO: Circumventricular organ
DCE-MRI: Dynamic contrast-enhanced magnetic resonance imaging
DTPA: Diethylenetriaminepentaacetic acid
ECS: Extracellular space
FITC: Fluorescein isothiocyanate
Gd: Gadolinium
GlySar: Glycylsarcosine
IAP: Iodoantipyrine
IGF1: Insulin-like growth factor-1
IOI: Integrated optical imaging
MMCA: Macromolecular magnetic resonance contrast agent
MR: Magnetic resonance
MRCA: Magnetic resonance contrast agent
MRI: Magnetic resonance imaging
MW: Molecular weight
NCI: National Cancer Institute
PS: The capillary permeability coefficient and the surface area product
QAR: Quantitative autoradiography
RBC: Red blood cells
RISA: Radioiodinated serum albumin
ROI: Region of interest
RTI: Real time iontophoretic
RTP: Real time pressure
SDI: Step-down infusion
SE: Spin echo
TIFP: Tumor interstitial fluid pressure
TMA: Tetramethylammonium
VTA-GE: Variable tip angle gradient echo.
